# Transmural healing in ulcerative colitis patients improves long-term outcomes compared to endoscopic healing alone

**DOI:** 10.1093/ecco-jcc/jjaf149

**Published:** 2025-08-22

**Authors:** Chong-Teik Lim, Christoph Teichert, Maarten Pruijt, Floris De Voogd, Geert D’Haens, Krisztina Gecse

**Affiliations:** Department of Gastroenterology and Hepatology, Amsterdam University Medical Centers, Amsterdam 1081 HV, The Netherlands; Department of Gastroenterology and Hepatology, Singapore General Hospital, 168753, Singapore; Department of Gastroenterology and Hepatology, Amsterdam University Medical Centers, Amsterdam 1081 HV, The Netherlands; Department of Gastroenterology and Hepatology, Amsterdam University Medical Centers, Amsterdam 1081 HV, The Netherlands; Department of Gastroenterology and Hepatology, Amsterdam University Medical Centers, Amsterdam 1081 HV, The Netherlands; Department of Gastroenterology and Hepatology, Amsterdam University Medical Centers, Amsterdam 1081 HV, The Netherlands; Department of Gastroenterology and Hepatology, Amsterdam University Medical Centers, Amsterdam 1081 HV, The Netherlands

**Keywords:** transmural healing, ulcerative colitis, intestinal ultrasound

## Abstract

**Background & Aims:**

Endoscopic healing (EH) is recognized as a long-term treatment goal for patients with ulcerative colitis (UC). We investigated whether transmural healing (TH) in UC as assessed by intestinal ultrasound (IUS) is associated with improved outcomes compared to EH alone.

**Methods:**

We performed a retrospective study in a tertiary center on patients with left-sided or extensive UC on stable maintenance treatment who had EH [Mayo Endoscopic Subscore (MES) ≤1) and an IUS performed within 6 months of an endoscopy with no treatment alterations between IUS and endoscopy. TH was defined as bowel wall thickness (BWT) <3 mm. The primary outcome was relapse-free survival in patients with and without TH.

**Results:**

A total of 61 patients (MES 0: 44.3%; MES 1: 55.7%) with a median follow-up of 20 months were included. On IUS, 72% of patients had TH. Twenty-three patients had a relapse (first-year relapse risk: TH: 7.5% vs no TH: 29.4%, *P* = .004; MES 0: 3.7% vs MES 1: 20.8%, *P *= .059). In multivariate Cox regression, female gender [hazard ratio (HR), 2.63; 95% CI 1.05–6.58; *P *= .039], two or more previous advance therapies (HR, 4.06; 95% CI 1.08–15.28; *P *= 0.038), and non-TH (HR, 3.99; 95% CI 1.31–12.20; *P *= .015) were associated with a relapse whereas EH level (MES 0 vs MES 1) was not an associated factor (HR, 1.06; 95% CI 0.32–3.55; *P *= .924)

**Conclusions:**

In UC patients TH is associated with lower relapse risk compared to EH alone. These findings imply that IUS is a non-invasive, low-cost alternative to endoscopy for stratifying UC patients for risk of relapse.

## 1. Introduction

Inflammatory bowel diseases (IBD)—including Crohn’s disease (CD) and ulcerative colitis (UC)—are chronic diseases with a relapsing remitting disease course. The STRIDE-II consensus identified endoscopic healing (EH) as a long-term goal in the management of both UC and CD.^1^ Whilst not being a formal target, remission can be further evaluated by histology in UC and by cross-sectional imaging in CD. Interestingly, transmural healing (TH) was voted the least important goal in UC during the STRIDE-II consensus, as UC is traditionally viewed as a disease limited to the mucosa. However, previous studies have shown structural alterations not only in the mucosa, but also in the submucosa of UC patients.^2–4^ Moreover, the submucosa is the most responsive bowel wall layer on intestinal ultrasound (IUS) when anti-inflammatory treatment is started.^4^^,^^5^

IUS has emerged as a reliable tool for assessing disease activity in UC by mainly relying on the measurement of bowel wall thickness (BWT). Reduction and normalization of BWT correlates well with endoscopic response and remission.^5^ In contrast to conventional endoscopy, IUS can visualize all bowel wall layers.

In this study, we therefore aimed to explore the impact of TH on long-term outcomes in a real-world cohort of UC patients.

## 2. Materials and methods

### 2.1. Study design

We conducted an exploratory retrospective study of UC patients in the Amsterdam University Medical Centre (AUMC) after obtaining approval from the medical ethical committee (METC 2024.0236). All adult consecutive patients with UC between January 2017 and June 2024 were identified from the electronic medical record using the International Classification of Diseases 10th revision with Clinical Modification code ICD-10: K51.xx.

Patients were eligible for inclusion if they had (1) EH defined as Mayo Endoscopic Subscore (MES) ≤1 on the most severe segment on endoscopy, (2) an IUS performed within 6 months of endoscopy, (3) ongoing stable maintenance treatment without oral or intravenous corticosteroids for at least 2 months prior to endoscopy or at least 6 months prior to IUS (if it was performed first) and (4) a clinical follow-up of at least 6 months after endoscopy. Patients with ulcerative proctitis were excluded.

### 2.2. Data extraction

Demographic, clinical, biochemical, imaging, endoscopy and histological data were extracted manually from the electronic medical record system (EPIC, Verona, WI, USA). This included age, gender, disease duration, disease location, previous and current use of immunosuppressant therapy (aminosalicylates, immunomodulators, corticosteroids, and advanced therapies), dates of endoscopy, and imaging.

### 2.3. Endoscopic assessment

The AUMC is a tertiary referral center for IBD and thus all endoscopies were performed or supervised by one of eight IBD physicians who manage all IBD patients. Endoscopies were performed to evaluate treatment response in patients on maintenance therapy or if surveillance endoscopy was due. All standardized endoscopy reports were assessed for EH according to the MES on the most severe segment on colonoscopy or flexible sigmoidoscopy. If MES was not reported, MES 0 would be given if a description of normal or inactive disease was reported and MES 1 if mild disease, erythema, or decreased vascular pattern was described.^6^ Patients with endoscopies that had an incomplete report were excluded.

Where available, histological reports of biopsies were reviewed for the presence of histological activity. As standardized histological scoring indices were not routinely reported, histological healing was pragmatically defined as the absence of inflammation when no intra-epithelial neutrophils were described in the biopsy report.

### 2.4. Intestinal ultrasound assessment

All IUS scans were performed and reported by International Bowel Ultrasound Group certified practitioners who had >200 independent ultrasounds performed. IUS examinations were performed in the outpatient setting without fasting with a Philips EPIQ 5 machine using C5-1, L12-5, and L18-4 transducers or Samsung V8 machine using CA1-7, CA3-10, and LA2-14 transducers. Indications for IUS were for detection of disease activity, response to treatment, or routine monitoring.

IUS examinations were performed by systematically scanning all segments of the colon. IUS data on BWT, color Doppler signal (CDS), presence of fat wrapping, loss of colonic haustrations or wall layer stratification, and presence of reactive lymph nodes per colonic segment (ascending colon, transverse colon, descending colon, sigmoid) were collected from IUS reports. Presence of TH was defined as having a BWT<3 mm on all documented colonic segments. Image qualities and challenges in image acquisition were also reviewed and IUS results were only acceptable when a maximum of one non-sigmoid bowel segment (excluding the rectum) could not be assessed.

### 2.5. Definitions and outcomes

The primary outcome was relapse-free survival. UC relapse was defined as at least two of the following: Simple Clinical Colitis Activity Index of ≥5 or physician’s global assessment of relapse; fecal calprotectin ≥250 µg/g; and MES of ≥2 on any bowel segment.

The subsequent management of the relapses was reviewed. This included initiation of oral or intravenous corticosteroids or initiation, intensification or change of medications, hospitalization, and surgery. Patients were followed until the end of study on June 30, 2024.

### 2.6. Statistical analysis

Descriptive statistics were used to summarize the baseline characteristics, and categorical variables were presented as numbers and percentages whereas continuous variables were presented as means and SD or median and IQR when appropriate. A chi-square test and *t* test were used to compare the categorical and continuous variables respectively.

The time to UC relapse was defined as the time from endoscopy to UC relapse. Patients who had a UC relapse before the end of the study were considered as an event and censored. In addition, patients who have treatment non-compliance or intolerance or loss to follow-up were also censored at the last clinical evaluation.

The cumulative incidence rate of UC relapse was summarized using the Kaplan–Meier method and compared between groups using the log-rank test. Cox regression was used to evaluate the association between the variables found to influence time to relapse in univariate analysis. Collinearity diagnostics were performed using variance inflation factors (VIFs) prior to model fitting.

All variables with *P *< .10 on univariate analysis were integrated into the multivariate Cox-proportional model. A two-sided *P *≤ .05 was considered statistically significant. All data analyses were performed using SPSS version 28.0 (IBM Corp., Armonk, NY, USA).

## 3. Results

**Figure 1.**
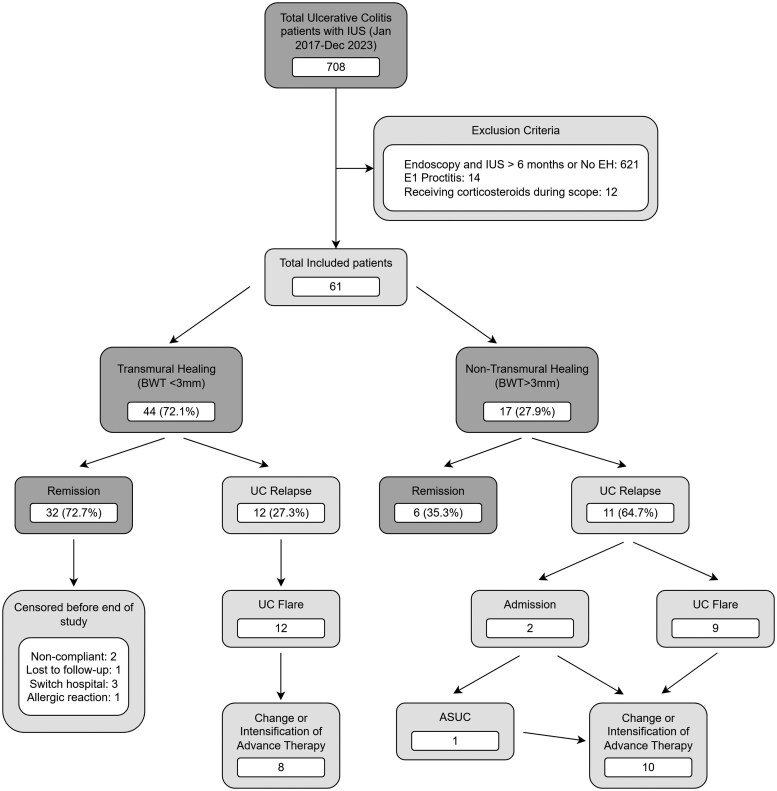
Flowchart of patients in the study. BWT, bowel wall thickness; EH, endoscopic healing; IUS, intestinal ultrasound.

A total of 708 UC patients with endoscopy and IUS data were identified with 61 fulfilling the inclusion criteria (Figure 1). Baseline characteristics are shown in Table 1 with 52.5% being male, a mean age of 43.5 ± 15.5 years, and a median disease duration of 9.8 years. The median time between endoscopy and IUS was 3 days (IQR −54 to 36) and subsequently patients were followed up for a median of 20.3 months (IQR 11.2–34.2).

**Table 1.** Baseline characteristics of patients.

Characteristic	Transmural healing (*n* = 44)	Non-transmural healing (*n* = 17)	Overall (*n* = 61)	*P*-value
**Gender** (male, %)	23 (52.3)	9 (52.9)	32 (52.5)	.96
**Duration of disease** (years, median, IQR)	12.0 (5.2, 20.3)	7.3 (4.2, 14.9)	9.8 (5.0, 17.4)	.13
**Age at baseline** (years, mean ± SD)	45.5 ± 15.0	38.3 ± 16.0	43.5 ± 15.5	.10
**White cell count** (×10^9^/L, median, IQR)	6.4 (4.7, 7.8)	6.1 (4.5, 7.4)	6.2 (4.6, 7.6)	.39
**Hemoglobin** (mmol/L, mean ± SD)	8.5 ± 1.0	8.1 ± 1.0	8.4 ± 1.0	.31
**Stool calprotectin** (µg/g, median, IQR)	31 (16, 153)	178 (93, 747)	87 (19, 185)	<.05
**C-reactive protein** (mg/L, median, IQR)	0.8 (0.4, 2.8)	1.1 (0.5, 6.7)	0.8 (0.4, 3.2)	.37
**Albumin** (g/L, median, IQR)	45.0 (40.0, 46.0)	44.5 (41.5, 45.3)	45.0 (41.0, 46.0)	.76
**Number of prior advanced therapy** (%)				.03
0	25 (56.8)	4 (23.5)	29 (47.5)	
1	9 (20.5)	6 (35.3)	15 (24.6)	
**≥**2	10 (22.7)	7 (41.2)	17 (27.9)	
**Previous anti-TNF use** (%)				.21
Yes	18 (40.9)	10 (58.8)	28 (45.9)	
No	26 (59.1)	7 (41.2)	33 (54.1)	
**Current medications** (%)				.06
5-Aminosalicylate	12 (27.3)	2 (11.8)	14 (23.0)	
Thiopurines/methotrexate	4 (9.1)	2 (11.8)	6 (9.8)	
Biologics	21 (47.7)	6 (35.2)	27 (44.3)	
JAK inhibitors	7 (15.9)	7 (41.2)	14 (23.0)	
**UC extent (Montreal classification)** (%)				.94
E2 Left-sided UC	16 (36.4)	6 (35.3)	22 (36.1)	
E3 Pancolitis	28 (63.6)	11 (64.7)	39 (63.9)	
**Endoscopic Mayo Score** (%)				<.05
0	27 (61.4)	0 (0)	27 (44.3)	
1	17 (38.6)	17 (100)	34 (55.7)	
**Time between endoscopy and IUS** (days, median, IQR)	15 (−81, 18)	0.0 (−6, 54)	3 (−54, 36)	.11
**Duration of follow-up** (months, median, IQR)	23.9 (13.3, 40.3)	14.0 (8.9, 31.3)	20.3 (11.2, 34.2)	.14
**Time from endoscopy to UC relapse** (months, mean ± SD)	20.9 ± 11.1	12.2 ± 7.8	16.7 ± 10.4	.04
	(*n* = 12)	(*n* = 11)		

IQR, interquartile range; IUS, IUS, intestinal ultrasound; SD, standard deviation; TNF, tumor necrosis factor; UC, ulcerative colitis.

**Figure 2.**
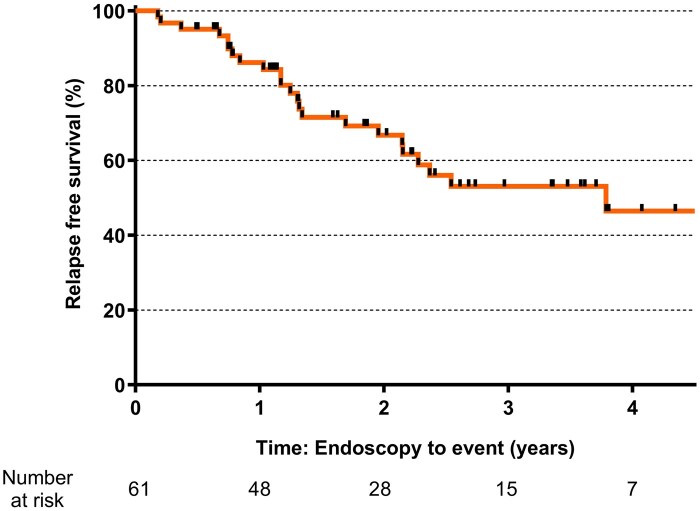
Kaplan–Meier estimates of overall cumulative risk of relapse survival.

The overall relapse-free survival of the total cohort was 86.2%, 66.8%, 53.1%, and 46.4% at the first, second, third, and fourth year respectively (Figure 2).

### 3.1. Transmural healing

**Figure 3.**
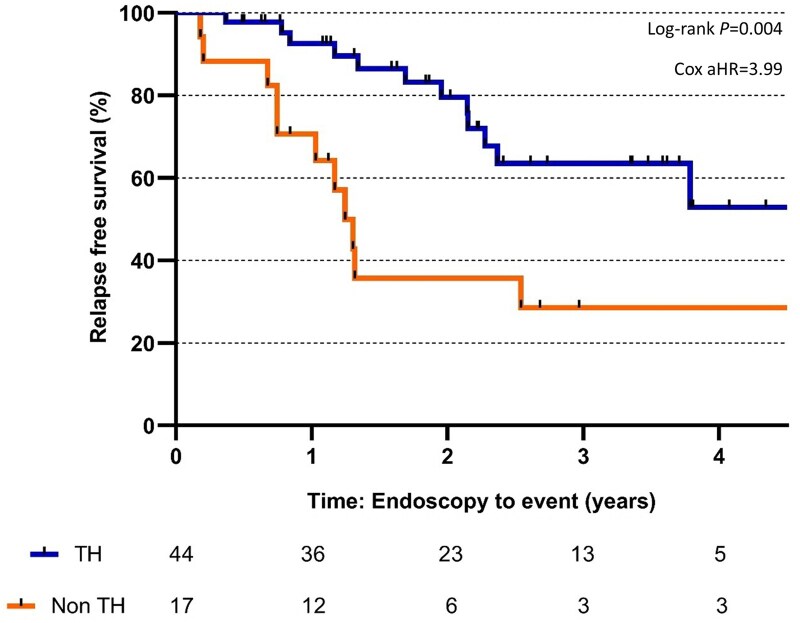
Kaplan–Meier estimates of relapse-free survival for transmural healing. TH, transmural healing.

At baseline, 44 patients had TH, with 27 of them (61.4%) having a MES of 0. During follow-up, 23 (37.7%) patients had a relapse. Patients who achieved TH had longer relapse-free survival compared to those without TH (log-rank test, *P *= .004; Figure 3).

The relapse risk for the first and second year was 7.5% and 20.5% for the TH group, respectively, compared to 29.4% and 64.3% for the non-TH group. Additionally, more than 50% of patients in the TH group remained relapse-free up to 4 years compared to the non-TH group. The mean time to the first relapse was 20.9 ± 11.1 months for the TH group and 12.2 ± 7.8 months for the non-TH group (*P *= .042). The median time between endoscopy and IUS for the TH and non-TH group was not associated with relapse (*P *= .943 vs *P *= .416).

**Figure 4.**
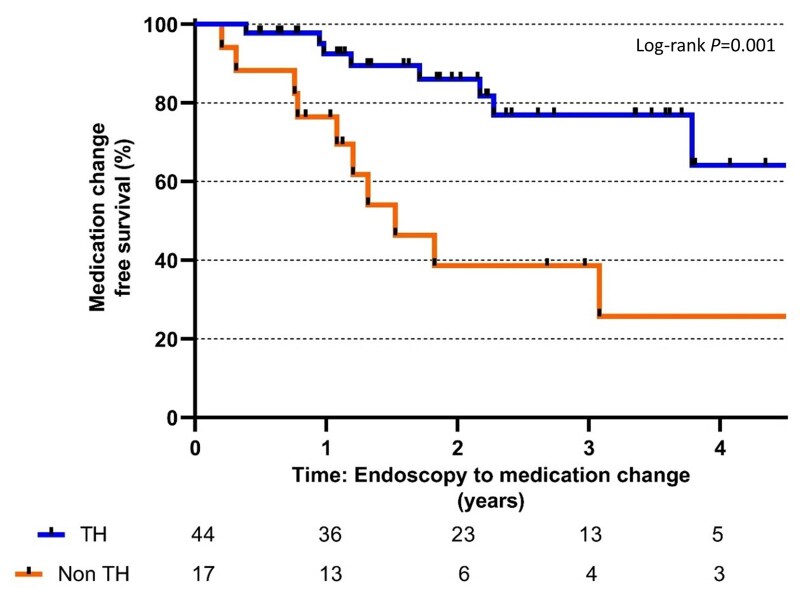
Kaplan–Meier estimates of medication change-free survival for transmural healing. TH, transmural healing.

Clinical symptoms and biochemical changes were the most common presentation of a relapse. Eight patients required the use of oral corticosteroids in addition to change of UC medications. Two patients from the non-TH group were admitted, and one was subsequently treated for an acute severe colitis. Following a relapse, eight patients from the TH group and 10 patients from the non-TH group underwent a change or intensification of advanced therapies. Patients who achieved TH had significantly longer medication change-free survival compared to those without TH (log-rank test, *P* = .001; Figure 4).

### 3.2. Endoscopic healing

The risk of relapse for MES 0 at the first year was lower than MES 1 (3.7% compared to 20.8%), although this was not statistically significant (log-rank test, *P *= .059; Supplementary Figure 1). When stratifying for MES, TH showed improved relapse-free survival for patients with MES 1 (log-rank test, *P *= .045; Supplementary Figure 2). Moreover, there was a trend towards shorter time to relapse for patients with MES 1 without TH compared to those with TH (12.2 ± 7.8 months vs 18.4 ± 7.6 months, *P *= .131).

**Figure 5.**
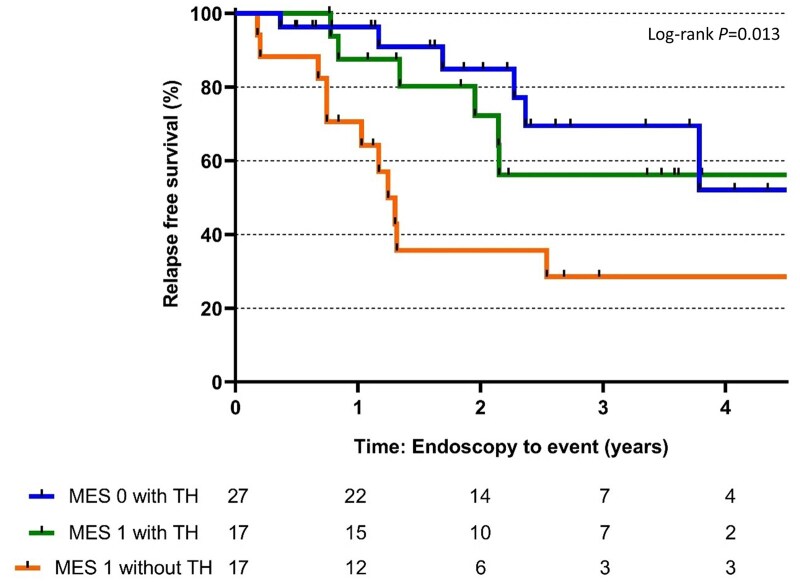
Kaplan–Meier estimates of relapse-free survival for endoscopic healing and transmural healing. MES, Mayo Endoscopic Subscore; TH, transmural healing.

The effect of achieving TH appears marginal when stratifying into MES 0 and 1 (*P *= .481). Although there is a reduced relapse risk at the first year for MES 0 with TH (3.7%) compared to MES 1 with TH (12.5%), the effect appears to be less profound after 4 years (log-rank test, *P *= .013; Figure 5).

### 3.3. Histological healing

Histological data were available for 40 patients, with 25 patients (10/13 MES 0, 15/27 MES 1) having histological healing. The presence of histological healing was not statistically significant for risk of UC relapse (log-rank test, *P *= .890). Further stratification of histological data among patients with MES 1 also did not show any statistical significance, although the first-year relapse risk for patients with histological healing was numerically lower (13.3% compared to 25.0%, log-rank test, *P *= .782; Supplementary Figure 3).

### 3.4. Clinical factors associated with UC relapse

Univariate Cox regression analysis showed female gender [hazard ratio (HR) 2.50 (95% CI, 1.06–5.92; *P *= .04)], previous use of two or more advance therapies [HR = 3.07 (95% CI, 1.17–8.10; *P *= .02)], and not achieving TH [HR = 3.17 (95% CI, 1.39–7.21; *P *= .01)] as factors associated with relapse (Supplementary Figures 4 and 5). These factors remain significant on multivariate analysis (collinearity diagnostics, all VIFs < 2.5) after correcting for age, extent and disease duration, medical therapy during endoscopy, and presence of EH (Table 2). In the subgroup multivariate analysis of patients with only MES 1, both female gender [hazard ratio, 4.16 (1.42–12.20); *P *= .01) and non-TH [hazard ratio, 3.81 (1.35–10.76); *P *= .01] were significant factors associated with UC relapse (Table 3).

**Table 2.** Predictors of variables associated with ulcerative colitis relapse.

Factor	Univariate analysis			Multivariate analysis		
	HR	95% CI	*P*	HR	95% CI	*P*
Gender	2.50	1.06–5.92	.04	**2.63**	**1.05–6.58**	**.04**
Age	0.99	0.96–1.02	.42			
Montreal classification (E2/3)	1.27	0.54–2.97	.58			
Duration of disease	1.00	1.00–1.00	.71			
Previous advance therapy						
0	1.00		.05	1.00		.02
1	1.19	0.41–3.42	.75	0.84	0.23–3.06	.79
≥2	3.07	1.17–8.10	.02	**4.06**	**1.08–15.28**	**.04**
Current medical therapy						
5-ASA	1.00		.35	1.0		.28
IMM/methotrexate	4.52	0.88–23.33	.07	3.35	0.56–20.00	.19
Biologics	2.06	0.57–7.49	.27	1.09	0.23–5.14	.91
JAK inhibitor	1.84	0.46–7.38	.39	0.61	0.10–3.56	.58
Endoscopic healing (MES 0/1)	2.39	0.94–6.06	.07	1.06	0.32–3.55	.92
Non-transmural healing (BWT > 3mm)	3.17	1.39–7.21	.01	**3.99**	**1.31–12.20**	**.02**

ASA, aminosalicylates; BWT, bowel wall thickness; CI, confidence interval; HR, hazard ratio; IMM, immunomodulator; JAK, Janus kinase; MES, Mayo Endoscopic Subscore; TH, transmural healing.

**Table 3.** Predictors of variables associated with ulcerative colitis relapse among MES 1.

Factor	Univariate analysis			Multivariate analysis		
	HR	95% CI	*P*	HR	95% CI	*P*
Gender	3.00	1.05–8.53	.04	**4.16**	**1.42–12.20**	**0.01**
Age	0.99	0.96–1.02	.49			
Montreal classification (E2/3)	1.89	0.61–5.83	.27			
Duration of disease	1.00	1.00–1.00	.75			
Previous advance therapy						
0	1.00		.36			
1	1.30	0.39–4.28	.67			
≥2	2.33	0.71–7.71	.17			
Current medical therapy						
5-ASA	1.00		.44			
IMM/methotrexate	3.75	0.62–22.87	.15			
Biologics	1.47	0.31–7.12	.63			
JAK inhibitor	1.34	0.26–6.92	.73			
Non-transmural healing (BWT > 3 mm)	2.68	0.98–7.29	.05	**3.81**	**1.35–10.76**	**.01**

ASA, aminosalicylates; BWT, bowel wall thickness; CI, confidence interval; HR, hazard ratio; IMM, immunomodulator; JAK, Janus kinase; MES, Mayo Endoscopic Subscore; TH, transmural healing.

## 4. Discussion

In the current study, TH in patients with UC was associated with longer relapse-free survival and this outcome was independent of the MES. Recent studies have focused on IUS in predicting treatment response or failure, correlation of BWT with histology or endoscopic activity, and transmural activity on risk of colectomy.^5^^,^^7–9^ However, there is a paucity of research on the clinical applicability of IUS in monitoring UC patients who are in clinical remission.

In this study, TH was defined as BWT < 3mm, consistent with current data suggesting a similar cut-off for CD,^10^ and in line with expert consensus supporting the applicability of this definition in UC.^11^ BWT < 3 mm has been shown to be highly correlated with colonic segments with endoscopic remission.^5^^,^^12^ The inclusion of CDS, bowel wall stratification, and potentially submucosal hyperechogenicity^13^ may further refine the identification of optimal markers for transmural remission in UC but no study has compared the superiority of the different definitions. Nonetheless, BWT remains the most reliable IUS parameter.^11^

Our findings suggest that achieving TH may lead to longer relapse-free survival than EH alone, regardless of whether patients had MES 0 or 1. According to a meta-analysis on EH, patients with MES 0 and 1 had an annual relapse risk of 14% and 29%, respectively.^14^ In comparison, our cohort demonstrated that achieving TH has an overall relapse risk of 7.5% during the first year. This incremental benefit in relapse-free survival when achieving TH is comparable to that of histological healing for patients with MES 0. In our cohort, patients with MES 0 and TH appear to have a 1-year relapse-free survival of 3.7%, which is comparable with pooled data from 10 studies for patients with MES 0 and histological remission (5%).^14^ Furthermore, in a smaller subgroup analysis of patients with MES 1, we showed that the first-year risk of relapse for MES 1 patients with TH is comparable to those with histological healing (12.5% vs 13.3%) but TH appears to have a more durable effect at reducing the risk of relapse at the second year (27.8% vs 48.7%).

The possibility of achieving TH in MES 1 has been demonstrated as a BWT < 3.2 mm was able to discriminate MES 0–1 and Mayo 2–3.^15^ This is an important finding to explore the utility of using TH to stratify risk of relapse among patients with MES 1, similar to studies which demonstrated the differences in risk of relapse for histological activity in MES 1 patients.^16^^,^^17^ Notably, among patients with MES 1, those who achieved TH experienced lower relapse rates compared to those without TH. However, this observation—along with comparisons of the clinical durability of transmural, endoscopic, and histological healing—requires validation in larger prospective cohorts, as the current study is exploratory in nature and not powered to assess these relationships definitively.

In our study female gender was a significant independent factor which was associated with UC relapse. Although we are unable to fully account for the role of gender in relation to a flare, one could hypothesize that women with IBD could employ a more proactive attitude, which could lead to more awareness and reporting of symptoms.^18^

We observed that the previous use of two or more advanced therapies was significantly associated with UC relapse. Our study population of tertiary-center referral patients with complex, treatment-refractory disease could contribute to this finding. Long-standing active UC is associated with several histological changes, including increased thickness of the lamina propria, hypertrophy of the muscularis mucosa, and submucosal fat deposition.^19^ These changes may contribute to the observed increase in BWT and hyperechogenicity on IUS,^13^ which may indicate a non-transmural healing pattern. Our cohort of non-TH patients have significantly higher exposure to advanced therapies compared to the TH group, probably reflecting a chronic active disease that is refractory to conventional therapy.

From our findings, TH is an indicator of long-term relapse-free survival in UC patients. A similar concept for TH has been explored in CD with current evidence suggesting an improved long-term outcome for patients with TH.^10^^,^^20^ IUS is a quick, well-tolerated procedure that does not require bowel preparation, and is cost-effective and environmentally friendly.^21–23^ These advantages of IUS make it an appealing option as a monitoring tool for UC.

The strengths of this study lie in the inclusion of patients with EH who were on stable medications, allowing us to study the effect of TH in a UC population that had already achieved their treatment targets. Additionally, all UC patients in our center who underwent IUS were reviewed for inclusion, which reduces selection bias. Lastly, the duration of follow-up (median: 20.3 months) provides valuable information on the durability of remission after achieving TH in patients with EH.

There are also some limitations to this study. First, our definition of TH is based solely on BWT and does not integrate CDS, fat wrapping, or haustration pattern. These are composites of the Milan ultrasound criteria and/or the UC IUS index.^15^^,^^24^ However, different definitions of TH have not been compared to evaluate long-term outcomes. BWT is the most reliable IUS parameter and therefore measuring BWT alone may be easier to adopt in routine clinical practice.^25^ Second, the procedure timing and interval between endoscopy and IUS could not be standardized due to the retrospective nature of this study. Ideally, a shorter interval between the investigations would be preferable to reduce monitoring bias among patients. However, our analysis shows the interval of the investigations was not significant between the TH and non-TH group. Furthermore, IUS and endoscopies were performed and reported as part of routine care before the study’s conception, which reduced reporting bias but resulted in the limitation of no blinding or central reading for these procedures. As this is a retrospective study, some degree of missing data is anticipated. To preserve the integrity of statistical analysis, patients with missing information that could compromise evaluation of the primary aim were excluded. Finally, our findings are only applicable to UC patients with left-sided or extensive colitis. Patients with a history of ulcerative proctitis only or endoscopic disease limited to the rectum were excluded due to known limitations of transabdominal ultrasonography in accurately measuring the BWT of the rectum because of its distance from the abdominal wall.^15^^,^^26^ However, transperineal ultrasonography, an emerging technique, could be a viable additional option for patients with disease limited to the rectum on endoscopy while also ensuring patients with residual proctitis are not missed.^27^

In conclusion, TH is associated with a lower risk of relapse in UC patients with EH and can be used to further stratify risk of relapse among patients with MES 1. IUS could therefore offer a non-invasive and cost-effective alternative to assess risk of relapse and guide de-escalation strategies in patients with UC.

## Supplementary Material

jjaf149_Supplementary_Data

## Data Availability

Data, analytic methods, and study materials will be made available to other researchers on reasonable request.
