# Supplementary material for: Transmural healing in ulcerative colitis patients improves long-term outcomes compared to endoscopic healing alone
Source: J Crohns Colitis. 2025 Aug 22;19(9):jjaf149. doi: 10.1093/ecco-jcc/jjaf149 (PMC12499911; doi:10.1093/ecco-jcc/jjaf149)
Supplement: jjaf149_Supplementary_Data [file jjaf149_supplementary_data.zip › Supplementary Data Revised.docx]

Supplementary Figure 1: Kaplan Meier Estimates of relapse free survival for endoscopic healing


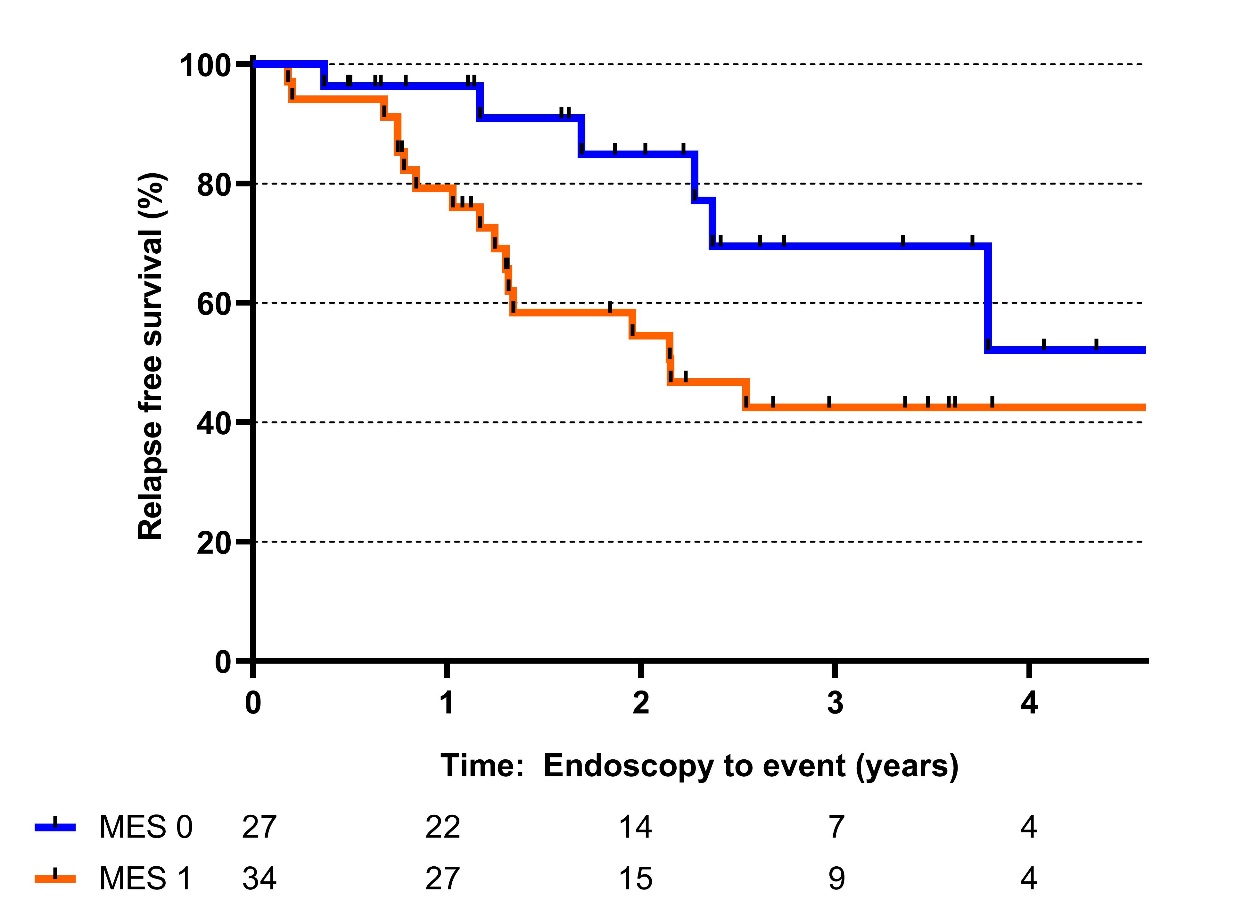


Log-rank *P*=0.059

Cox aHR=1.06

(95% CI: 0.32-3.55), *P*=0.92


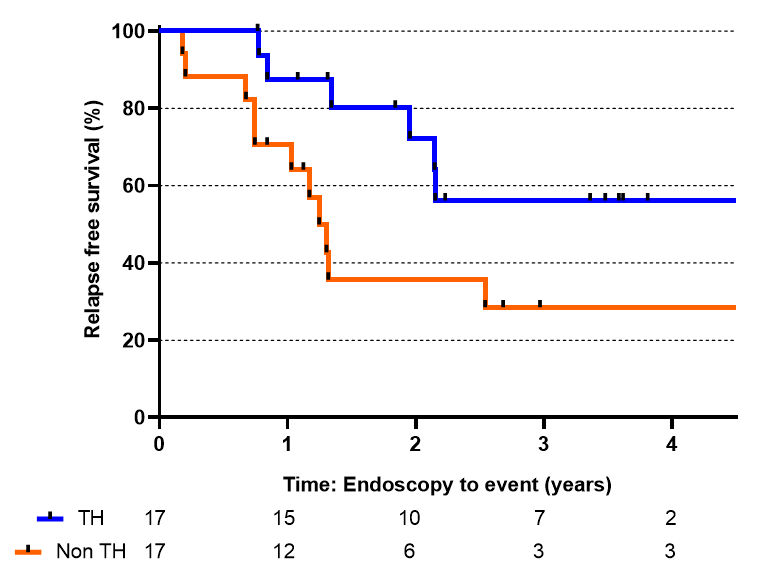
Supplementary Figure 2: Kaplan Meier Estimates of relapse free survival for transmural healing with MES 1

Log-rank *P*=0.045

Cox aHR=3.81

(95% CI: 1.35-10.76), *P*=0.01

Supplementary Figure 3: Kaplan Meier Estimates of relapse free survival for histological healing among MES 1


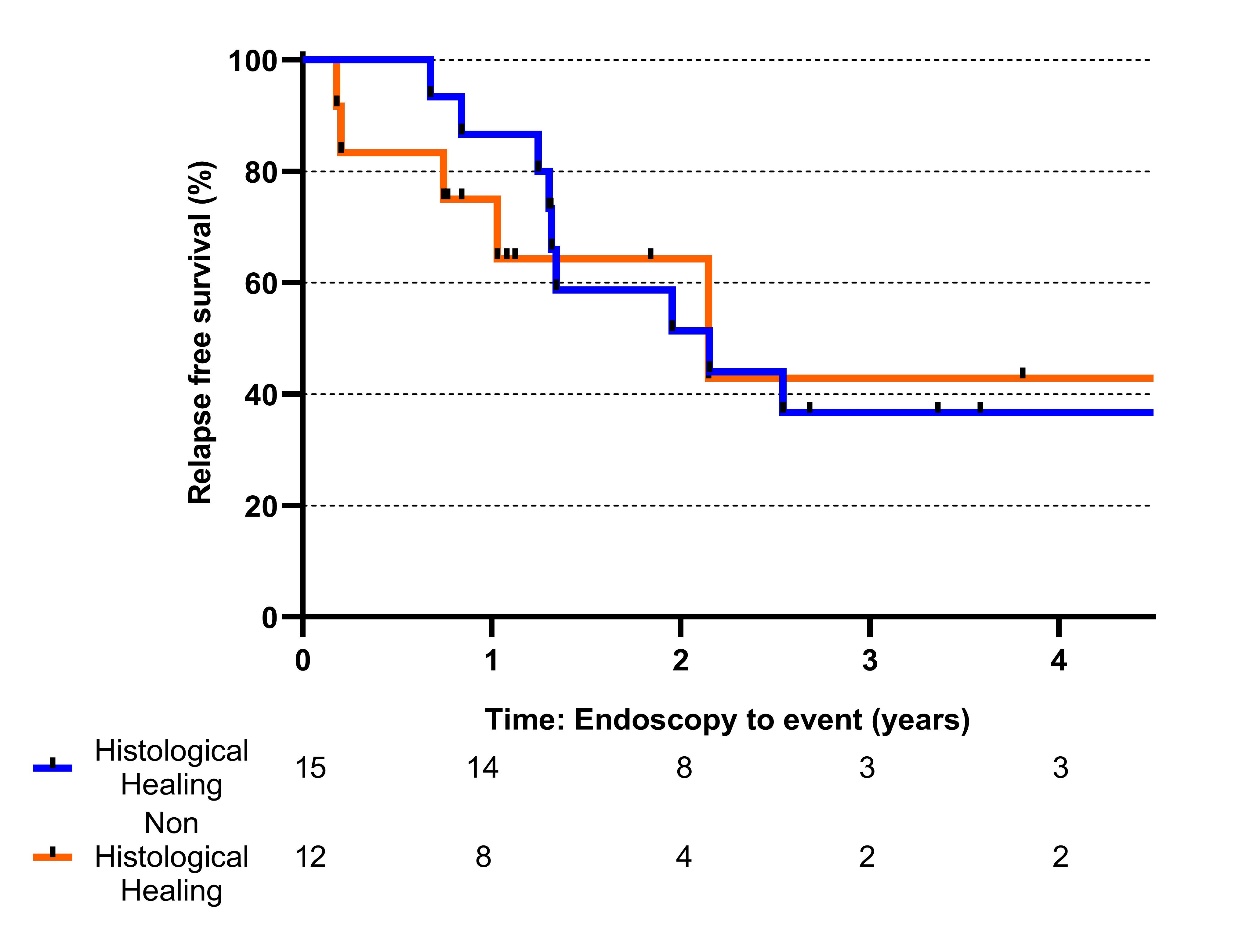


Log rank *P*=0.782

Supplementary Figure 4: Kaplan Meier Estimates of relapse free survival for gender


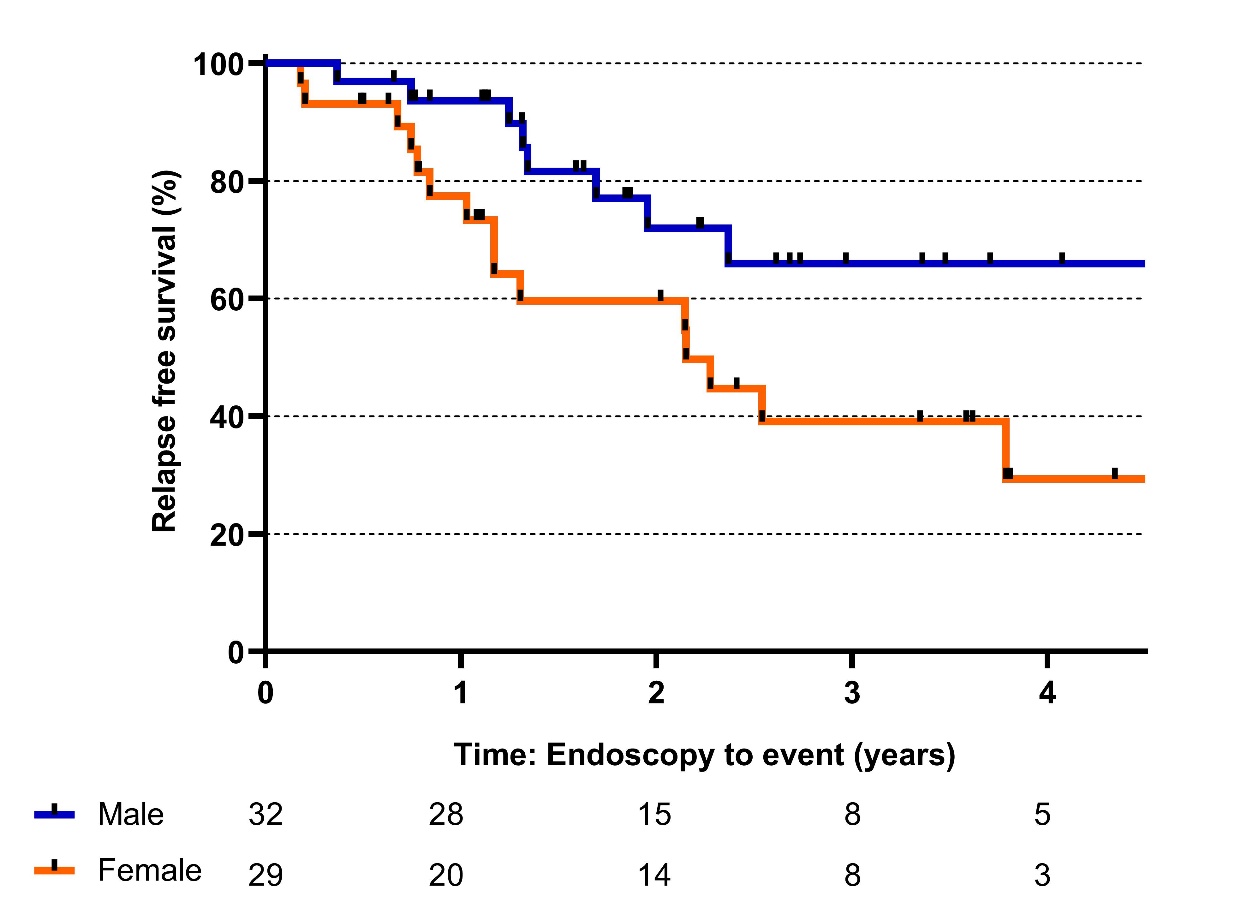


Log rank *P*=0.36

Cox aHR=2.63

(95% CI: 1.05-6.58), *P*=0.04

Supplementary Figure 5: Kaplan Meier Estimates of relapse free survival for number of previous advance therapies


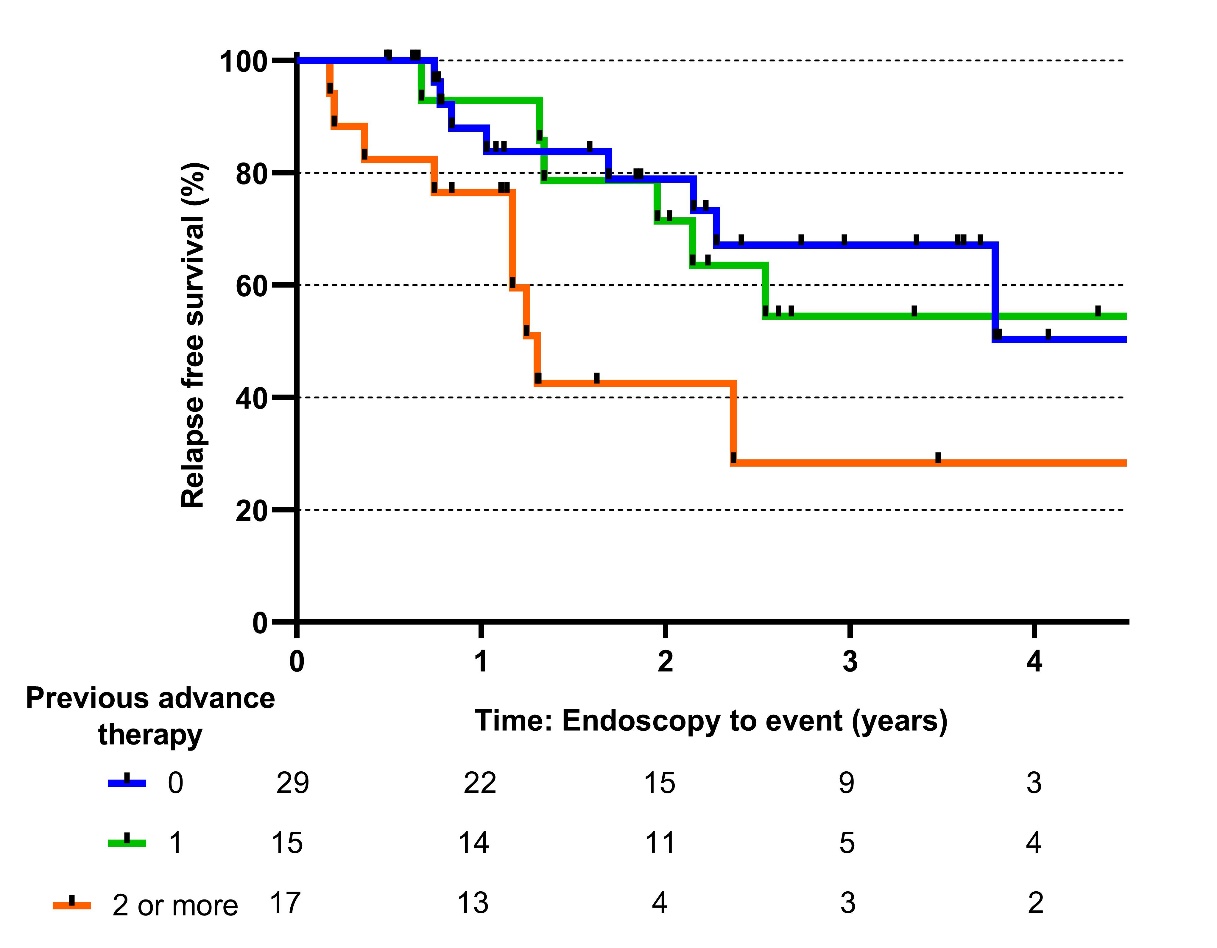


Log rank *P*=0.041

2 or more previous advance therapy

Cox aHR=4.06

(95% CI: 1.08-15.28), *P*=0.04
